# Fabrication of Three-Dimensional Microstructures on SiC Substrates by Using 355 nm Nanosecond Lasers: Process Control and Morphology Evolution

**DOI:** 10.3390/mi17070854

**Published:** 2026-07-17

**Authors:** Hsin-Yi Tsai, Yu-Hsuan Lin, Kuo-Cheng Huang, J. Andrew Yeh, Chen-Ju Lee

**Affiliations:** 1 National Center for Instrumentation Research, National Institutes of Applied Research, Hsinchu 300092, Taiwan; kellytsai@niar.org.tw (H.-Y.T.); marklin@niar.org.tw (Y.-H.L.); chengru@niar.org.tw (C.-J.L.); 2 Department of Power Mechanical Engineering, National Tsing Hua University, Hsinchu 300044, Taiwan

**Keywords:** silicon carbide (SiC), ultraviolet (UV) laser, 3D microstructure, morphology

## Abstract

Silicon carbide (SiC) has high thermal conductivity and thermal stability; however, its high hardness and brittleness make the fabrication of three-dimensional (3D) SiC microstructures—particularly those intended for thermal management of power devices—highly challenging. Because SiC exhibits strong absorption in the ultraviolet (UV) spectral range, this study conducted UV nanosecond laser irradiation to perform dry, direct-write processing on SiC, with material removal achieved through vaporization. It established an optimization workflow covering processes from the selection of planar processing parameters to the fabrication of 3D micropillar arrays with high surface quality and geometric fidelity. The key process variables were the pulse repetition frequency, nominal laser power, number of repeated scans per layer, and number of *Z*-direction focal shifts between layers. The micropillar arrays fabricated using the proposed approach were characterized in terms of their total material removal depth, sidewall verticality, and top-surface roughness. The results indicated that processing with a high repetition frequency resulted in favorable sidewall verticality; however, the pillar top surfaces were susceptible to high roughness resulting from spatter and melt backfilling. To address this problem, a strategy involving the fabrication of fewer shifting layers and the use of more scan repetitions per layer was employed. This strategy mitigated cumulative defocus errors, increased the total material removal depth, and achieved a suitable balance among removal depth, sidewall verticality, and top-surface roughness. Overall, this study provides practical guidelines for the direct-write 3D microstructuring of hard materials such as SiC. These guidelines have potential applications in the rapid fabrication of chip-level heat dissipation microstructures. They can reduce process complexity and manufacturing cost while improving design flexibility for 3D thermal architectures.

## 1. Introduction

Silicon-based power devices operated under high blocking voltage, temperature, and switching frequency are approaching their fundamental material-imposed performance limits. Compared with silicon, silicon carbide (SiC) has superior electrothermal properties, including a wider bandgap, a higher-strength critical breakdown electric field, and higher thermal conductivity [1]. Thus, with SiC, a thinner drift layer and higher doping concentration can be used for the same voltage rating, resulting in substantially lower specific on-resistance, supporting fast switching and high-temperature reliability [2], and improving the feasibility of high-frequency and high-power-density system designs. Consequently, SiC has emerged as a key replacement for silicon in high-frequency, high-power-density, and high-temperature applications, becoming a critical wide-bandgap semiconductor material for use in electric-vehicle traction inverters, charging infrastructure, renewable energy converters, and high-power supply units.

Technological bottlenecks corresponding to power modules and applications involving high heat flux density extend from the wafer material to the packaging and interfacial layers. However, three-dimensional (3D) arrayed microstructures—such as microchannels, micropillars, microfins, microgrooves, and pore and via arrays—can increase the effective heat dissipation surface area without substantially increasing the device volume. They can also enhance convection and boiling heat transfer through liquid- or air-cooling modules [3,4]. Moreover, the thermal resistance of and accumulation of heat in power components can be reduced by controlling the surface wettability and capillary regeneration of microstructures, thereby improving the power density and reliability of the components. In particular, studies have demonstrated the ability of SiC microchannel cooling to reduce heat flux, with the microstructured SiC substrate serving as a heat dissipator with high thermal conductivity, high temperature tolerance, and favorable thermal expansion compatibility. Cassada et al. [5] investigated the variation in thermal resistance with flow rate, microchannel aspect ratio, and hydraulic diameter for two microchannel geometries. Their experiments indicated that microchannels with a high aspect ratio and small hydraulic diameter achieved extremely low thermal resistance under high thermal loading. Specifically, when the heater temperature was below 60 °C, the thermal resistance between the heater surface and fluid inlet was as low as 0.024 cm^2^·°C/W, indicating highly efficient heat dissipation for high-power, high-density electronic devices. Yang et al. [6] employed plasma etching to fabricate microchannel arrays with a channel width of 15 µm and a depth of 100 µm on SiC substrates and then evaluated the thermal performance of a SiC manifold microchannel heat sink under various background heat flux densities and localized hotspot heat flux conditions. Their results indicated effective removal of a background heat flux of 3190 W/cm^2^ over an area of 5 mm × 5 mm, with a pressure drop of only 32 kPa. Moreover, the SiC manifold microchannel heat sink could remove a localized hotspot heat load of 12.0 kW/cm^2^ within a region of size 100 µm × 100 µm while limiting the hotspot temperature rise to smaller than 150 °C.

The approaches most commonly adopted for fabricating 3D microstructures on SiC rely on inductively coupled plasma reactive ion etching (ICP-RIE) combined with hard masks to achieve anisotropic etching [7,8]. Typical strategies include adding oxygen to SF_6_-based plasma to enhance vertical etch profiles as well as investigating how variations in reactive ion etching and inductively coupled plasma power influence etch selectivity for SiC, thereby enabling the formation of 3D features with various geometries and supporting the development of high-aspect-ratio processes. However, plasma-based dry etching of SiC has several intrinsic limitations, including stringent mask selectivity requirements, relatively low etch rates, high sidewall roughness of the etched microstructures, and difficulties in continually controlling morphology for high-aspect-ratio patterns [9]. Studies have increasingly used femtosecond or other ultra-short-pulse lasers to directly etch SiC surfaces for the fabrication of micropillars, hole arrays, and microgrooves [10]. In addition, scholars have reported hybrid micromachining approaches in which laser processing is employed to generate a coarse preform, following which ICP-RIE is conducted to refine the geometry, improve dimensional fidelity, and reduce surface roughness, thereby enhancing overall structural accuracy and surface quality [11,12].

In direct material removal, femtosecond lasers induce nonlinear absorption and ultrafast deposition, thereby substantially suppressing thermal diffusion effects. Therefore, femtosecond lasers are particularly suitable for fabricating high-precision, low-thermal-damage 3D microstructures on SiC, a material that is hard, brittle, and chemically inert [13]. Moreover, high morphological flexibility can be achieved by tailoring the scan trajectory and adopting a layer-by-layer (slicing) strategy; however, this approach has limitations including relatively low throughput and debris redeposition. In contrast to femtosecond laser processing, nanosecond laser processing is dominated by thermally driven ablation mechanisms, benefits from mature equipment platforms, is inexpensive, and has high processing efficiency; thus, this method is attractive for rapidly producing microgrooves, microholes, and arrayed protrusion–depression textures. However, the drawbacks of nanosecond laser processing include the formation of pronounced heat-affected zones (HAZs) and recast layers, oxidation, and microcracking-related surface damage [14]. Table 1 summarizes the analysis of the cost, time, fabrication location, and application scenarios of 3D structures using ICP/RIE, femtosecond laser, and nanosecond laser. The advantages that this paper can currently propose and confirm are maskless operation, vacuum-free process, high flexibility in pattern switching, and the ability to quickly change 3D structural designs. 

Accordingly, to prevent the formation of defects, the laser processing parameters—such as fluence, pulse overlap, and scanning strategy—must typically be optimized, and appropriate postfabrication treatments are required. Nevertheless, nanosecond lasers have been widely adopted for SiC micromachining, including microstructure fabrication (e.g., cantilever fabrication) [15], drilling [16], and through-hole creation. Therein, Shi et al. [15] proposed a UV laser micromachining method for releasing SiC cantilever beams, as well as fabricating proof-mass and through-hole structures by laser ablation. They demonstrated that the pulse repetition frequency (PRF) and the number of machining cycles significantly affect the surface morphology and the extent of thermal effects. After optimization, a pulse repetition frequency of 35 kHz, four machining cycles, and a scanning speed of 200 mm/s were adopted for through-hole fabrication, providing a balance between machining quality and reduced thermal damage.

Tsai et al. [17] employed an ultraviolet (UV) nanosecond laser for dry material removal from SiC substrates, achieving an ablation depth of approximately 2 μm per pass. In the work, the effects of laser spot overlap on the compositional variation and hardness change of SiC were further investigated using EDS and nanoindentation measurements. They reported relatively low surface roughness when the laser spot overlap ratio was 30–60% and discovered that the SiC hardness after laser ablation could be reduced to less than 3% of its initial value. Zhang et al. [18] used a UV nanosecond laser to modify the surface of 4H-SiC and investigated the effects of laser fluence, scanning speed, and spot overlap on surface roughness. Their results indicated that increasing the fluence increased the oxygen content but reduced the carbon content at the processed surface, leading to a thicker oxide layer. Refs. [15,17,18] and the novelty of the present study are summarized in Table 2.

In addition, decreasing the scan speed and increasing the overlap ratio caused an increase in the oxygen content and a decrease in the SiC hardness. Bustamante et al. [19] employed an infrared nanosecond laser to fabricate high-aspect-ratio through-holes (aspect ratio of up to 17.10) through a multistep processing strategy while controlling microcracks and sizes of the HAZs to improve machining precision. Kim et al. [20] used a near-infrared nanosecond laser to create pyramid-shaped fin array microstructures on 4H-SiC and evaluated their boiling heat transfer performance through pool boiling experiments conducted at 5 °C. Because of laser-induced oxidation, the fabricated microstructures exhibited higher wettability and a smaller contact angle, which substantially promoted bubble nucleation and increased the heat transfer coefficient. To prevent laser-induced oxides from hindering material removal during SiC processing, Zhang et al. [21] used a blowing-assisted laser processing approach and compared the groove depths and widths achieved with versus without gas blowing. Their experimental results indicated that gas blowing enabled the fabrication of deeper, narrower, and higher-quality arrayed microgroove structures. Furthermore, Zhang et al. [22] adopted laser waterjet-assisted micromachining to produce high-aspect-ratio microgrooves on SiC. By cooling the processing zone and reducing thermal shock effects, this approach mitigated recast formation and microcracking while reducing surface roughness. Through the aforementioned approach, Zhang et al. [22] fabricated high-quality microgrooves with an aspect ratio of 3.66.

The aforementioned studies have mainly focused on using femtosecond lasers to fabricate 3D microstructures, employing UV nanosecond lasers to remove SiC before performing surface quality and hardness analyses, using near-infrared nanosecond lasers to develop pyramidal fins or through-holes, or employing air-blowing or water-assisted laser processing to create high-aspect-ratio microstructures. However, the use of UV nanosecond lasers for fabricating 3D microstructures on SiC, particularly the effects of laser parameters on microstructural surface characteristics and geometric profiles, remains insufficiently explored. Therefore, in this study, a UV nanosecond laser was used to fabricate 3D micropillar arrays on SiC. The effects of key processing parameters—including laser power, pulse repetition frequency, scanning speed, and number of repeated scans—on the material removal depth and the sidewall verticality and top-surface roughness of the fabricated 3D micropillar arrays were examined to obtain an optimized parameter set for micropillar array fabrication. The proposed method is a low-cost, maskless, consumable-free, and flexible direct-write approach for fabricating 3D microstructures on SiC. It has advantages such as pattern tunability and excellent surface morphology control. The proposed method can be used to fabricate heat-dissipating microstructures for SiC-based power semiconductor devices, thereby substantially improving their thermal management performance, reducing their fabrication cost, and expanding the application of SiC. Moreover, blowing assistance can be regarded as an integrable module for future work to further reduce redeposition and backfilling, thereby improving surface quality. The post-treatment processes, such as light CMP, wet/dry cleaning, oxide-layer removal, annealing, or subsequent metallization, can also be incorporated in future work. Further reliability validation, including fluid shear strength, thermal resistance, and thermal cycling tests, will also be required.

## 2. Fundamental Theory

In nanosecond laser ablation of SiC, high photon energy is applied to the SiC surface within a short pulse duration, leading to a high energy density, which results in rapid heat generation and consequently in material removal. For UV irradiation at 355 nm, the photon energy is approximately 3.49 eV, whereas the bandgap of 4H-SiC is approximately 3.26 eV. Therefore, incident photons can directly excite electrons from the valence band to the conduction band through interband absorption [23,24]. This process results in a higher absorption coefficient than can be achieved with an infrared laser, and the laser energy can be absorbed and converted into heat within a shallow near-surface region. The consequent localized temperature rise can induce melting, vaporization, and plasma formation [25], leading to the removal of material and the generation of molten ejecta. When the fluence approaches the ablation threshold, ultra-thin-layer removal may be accompanied by surface modification; therefore, UV nanosecond laser processing of SiC can simultaneously enable material ablation, surface oxidation, and functional modification.

**Figure 1:**
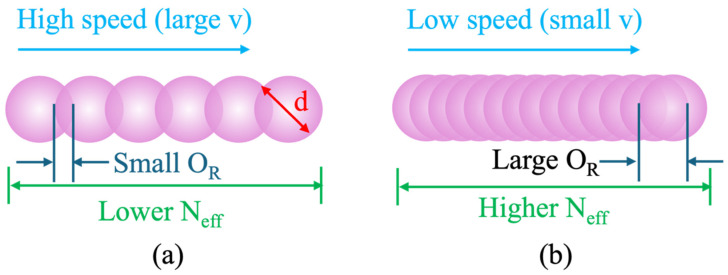
Schematic of the relationship between the overlap ratio (O_R_) and effective number per processed location (*N*_eff_) with (**a**) high and (**b**) low scanning speed in the *X*-direction.

**Figure 2:**
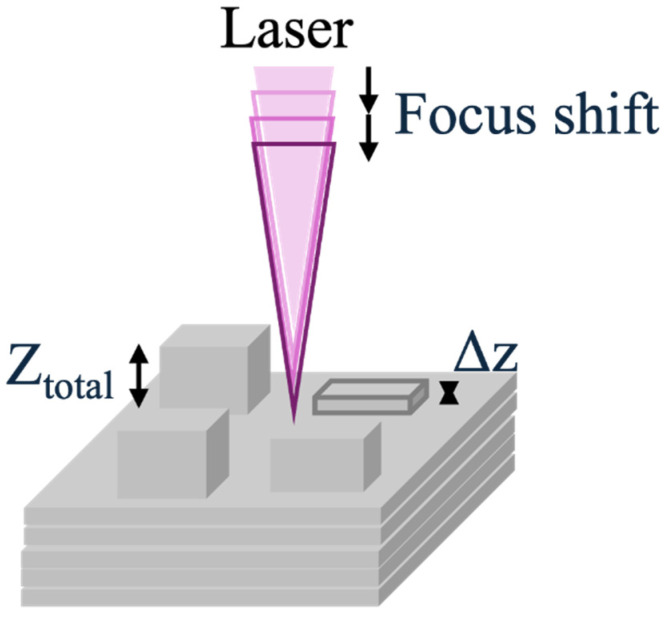
Schematic of the stacking of layers along the *Z*-axis.

During multipulse processing, pulse overlap and heat accumulation can reduce the effective multipulse ablation threshold; this is commonly referred to as the incubation effect [26,27] and may lead to ablation depth saturation [28]. Consequently, processing parameters such as the fluence, laser power (w), pulse repetition frequency (f), scanning speed (v), spot diameter (d), pulse overlap ratio (O_R_), and hatch spacing influence the effective number of pulses per processed location (N_eff_) and the accumulated laser energy [29,30,31]. The relationship among *O*_R_, the bite size (B_s_), and *d* is presented in Equation (1). Moreover, B_s_ and N_eff_ are expressed in Equations (2) and (3), respectively. Figure 1 displays a schematic of the relationship among between O_R_ and N_eff_. The processing parameters affect the material removal efficiency, sidewall morphology, occurrence of melt redeposition, and HAZ characteristics. On the basis of these mechanisms, multilayer fabrication of 3D microstructures typically begins with ablation over a single plane to form one layer, following which repeated scanning is conducted to stack N layers in the *Z*-direction (Figure 2). Through cumulative processing, the target overall depth (*Z*_total_) and the desired 3D profile can be achieved [Equation (4)]. To fabricate micropillar arrays, an inverse patterning strategy is adopted in which surrounding material is removed while the pillars are retained. Multilayer processing is then employed to control key geometric metrics, including the pillar’s verticality, squareness, and sidewall roughness. Because interactions between a UV nanosecond laser and SiC often involve surface oxidation and a reduction in near-surface hardness, O_R_ and the multilayer scanning strategy are strongly influenced by surface quality, processing efficiency, and defect formation (e.g., microcracks and recast layers). This study systematically investigated the effects of various laser parameters on microstructural morphological characteristics to enable the fabrication of high-quality 3D microstructures on SiC.

(1) $$O_{R}=\frac{d-B_{s}}{d}\times 100\%$$

(2) $$B_{s}=\frac{v}{f}$$

(3) $$N_{eff}=\frac{f\times d}{v}=\frac{d}{B_{s}}$$

(4) $$Z_{total}=N\times ∆z$$

## 3. Materials and Experimental Setup

### 3.1. Materials

SiC is a covalent compound consisting of silicon and carbon atoms with strong *sp*^3^ bonding [32]. It exhibits polytypism, which refers to multiple crystallographic phases arising from different sequences of stacking of atomic bilayers with the same chemical composition. Common SiC polytypes include the cubic form 3C-SiC and the hexagonal forms 4H-SiC and 6H-SiC. Although 4H-SiC and 6H-SiC have several similar advantageous properties—such as high hardness, excellent chemical corrosion resistance, and high thermal conductivity—their distinct stacking periodicities and sequences lead to differences in bandgap, carrier mobility, and anisotropic thermal transport. Therefore, in power device and high-heat-flux applications, the choice of polytype can directly influence the performance of the substrate and the effectiveness of the thermal interface. The thermal conductivity of SiC is primarily influenced by its crystallographic anisotropy (i.e., differences between thermal transport along the *c*-axis and within the basal plane). However, in practical applications, differences in thermal conductivity may arise between the Si face and the C face on account of damaged near-surface or subsurface layers, internal defects, residual stress, and interfacial thermal boundary resistance, all of which can alter the heat flow across the surface or bonded interfaces. Qian et al. [33] conducted the first systematic measurements of the thermal conductivity along the in-plane (basal-plane) and cross-plane (*c*-axis) directions in 4H-SiC and 6H-SiC. Their results indicated the following order in terms of thermal conductivity: semi-insulating 4H-SiC > n-type 4H-SiC > semi-insulating 6H-SiC. Wei et al. [34] investigated the variations in the thermal conductivity of 4H-SiC with doping level and temperature, noting that heat transport in this material was strongly influenced by temperature. Moreover, Deng et al. [35] quantified the thermal boundary resistance at the 4H-SiC/SiO_2_ interface and demonstrated that interfacial structures and defects can substantially affect heat transfer; thus, thermal boundary resistance is a critical factor in the design and fabrication of microstructured thermal interfaces.

Considerable polarity-related differences in the mechanical properties of the Si and C faces of 4H-SiC have been reported. Osipov et al. [36] compared the near-surface hardness of these faces through nanoindentation tests. They noted that within an indentation depth of 0–60 nm, the average hardness of the C face was approximately 1.5 times higher than that of the Si face. They also reported that the Young’s modulus of the C face was close to that of bulk 4H-SiC (~400 GPa) and approximately 2.3 times higher than that of the Si face. Wang et al. [37] conducted experiments and simulations to examine the damage mechanisms induced by material removal and grinding on the Si and C faces. The C face was discovered to generally exhibit higher hardness and have a higher elastic modulus but lower fracture toughness. In addition, the C face was found to be susceptible to high-density dislocations, and stress release in the processed region of the C face reduced grinding forces and surface roughness.

In SiC power semiconductor applications, the Si face is typically reserved for epitaxial growth and front-side device fabrication, whereas the C face serves as the primary heat conduction pathway and the bonding interface for packaging and heat spreader attachment. Consequently, constructing 3D thermal microstructures—such as microchannels, micropillars, and fin arrays—on the C face is expected to be beneficial for improving heat dissipation and reducing system-level thermal resistance. Accordingly, the present study conducted laser microstructuring experiments on the C face of 4H-SiC. Since it is an as-cut wafer that has not been polished, its average surface roughness of the unprocessed SiC is 0.182 µm.

### 3.2. Experimental Setup

#### 3.2.1. System and Parameters

This study employed a 355 nm solid-state Q-switched UV laser (AVIA NX 355-20, Coherent Inc., Santa Clara, CA, USA) with a maximum output power of 20 W. A short-focal-length focusing configuration was adopted to fabricate 3D microstructures on SiC because SiC has high hardness and a relatively high ablation threshold. Specifically, a high-NA (numerical aperture), short-focus lens was used to reduce the focal spot size and thus increase the laser fluence, thereby enabling efficient ablation of SiC. However, because a short-focus lens provides a limited depth of focus, precise *Z*-axis positioning and layer-by-layer focal adjustment were required during multilayer processing to ensure effective interaction between the laser and SiC. A two-axis galvanometric scanner was used to control the *XY* scanning trajectories within the patterned region, and an electrically actuated lens stage enabled *Z*-axis adjustment of the focal position. This experimental laser system integrated a telecentric focusing lens with a focal length of 109 mm, whose theoretical spot diameter at the focal point was approximately 10 µm.

In a galvanometer scanning system, the focal plane displacement is linearly related to the galvanometer deflection angle. Therefore, for a given galvanometer deflection angle, the size of the scanned region is proportional to the effective focal length of the lens. In this study, the calibration parameters of a lens with a focal length of 580 mm were used to convert the length (in millimeters) into a galvanometer deflection angle (in degrees). If the focal length is changed to 109 mm without the corresponding calibration settings being updated, the processing dimensions scale proportionally with the focal length, resulting in an approximately fivefold reduction in these dimensions. Therefore, the dimensions of all pattern geometries were prescaled by a factor of 5 in the design stage to ensure that the fabricated microstructures had the target dimensions. The maximum scan area of the laser system is limited by the scanning mirror and focusing lens configuration. After completing the software calibration update in the future, the single-scan processing area is expected to be approximately 60 × 60 mm. For areas exceeding this scanning range, stage stitching or repositioning will be required to complete large-area processing.

**Figure 3:**
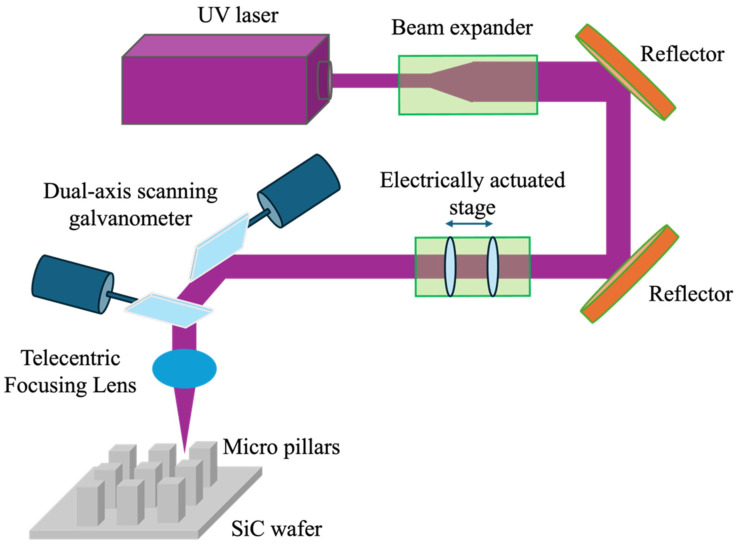
Schematic of the laser micromachining system used to fabricate three-dimensional (3D) microstructures on SiC.

The scanning speed during laser processing was optimized to 2.5 m/s. The laser power was varied from 10% to 100%, and the pulse repetition frequency was varied from 10 to 100 kHz. The single-pass ablation depth and surface characteristics (e.g., surface roughness) were measured under these conditions to establish an optimized parameter database. To evaluate 3D geometry formation, a 3 × 3 array pattern was fabricated using a 30-layer processing strategy. The effects of the laser power and pulse repetition frequency on the resulting 3D micropillar structures were assessed in terms of the depth, sidewall verticality, and surface roughness of the fabricated micropillars. To increase the achievable structure height through multi-layer processing, the morphological differences between repeated passes within a single layer and repeated processing over the entire multilayer sequence were analyzed. A schematic of the laser micromachining system for SiC microstructure fabrication is presented in Figure 3. The laser specifications and experimental parameters are summarized in Table 3.

#### 3.2.2. Structural Morphology Analysis

A 3D confocal laser scanning microscope (KEYENCE Inc., Osaka, Japan, VK-X200) was used to quantify the ablation depth and surface roughness after laser processing. In addition, it was employed to evaluate the geometrical fidelity of the fabricated micropillar arrays, including the sidewall angle and the surface roughness of top surface and ablated region. The confocal microscope, which was equipped with a high-magnification objective lens (20×), enabled noncontact and precise characterization of the overall geometry and detailed surface features of laser-fabricated 3D microstructures on SiC. After each set of processing parameters was completed, multiple data points (>3) were collected from different locations within the processed area. The results are presented as the mean value with the corresponding standard deviation.

#### 3.2.3. Experimental Process

The experimental process involved the following steps:

1. Ablation parameter setup: A SiC substrate was placed in the laser micromachining system and positioned at the focal plane of the telecentric focusing lens, with the C face oriented upward as the processing surface. The laser parameters—the laser power, pulse repetition frequency, and scanning speed—were configured through a system control interface (Table 3).
2. Process parameter optimization for single-layer fabrication: The single-layer fabrication process was first optimized by examining the relationship between the scanning speed and horizontal pulse overlap ratio when the pulse repetition frequency and laser power were fixed. A scanning speed corresponding to an overlap ratio of approximately 50% was selected as the default condition whenever feasible. Next, with the scanning speed and pulse repetition frequency held constant, the laser power was varied to quantify the single-pass ablation depth and corresponding surface roughness. An appropriate power level was then selected, and the pulse repetition frequency was varied to evaluate how the pulse energy density influenced the ablation depth and surface characteristics.
3. Examination of 3D fabricated structures: A 3 × 3 array pattern was designed and fabricated using a continuous 30-layer processing strategy. The integrity of the 3D structures obtained under different laser powers but a fixed scanning speed was examined to determine whether excessive energy input led to microstructure collapse or degradation. Subsequently, under a suitable laser power, the pulse repetition frequency was varied to assess key geometric and surface properties of the micropillars, including their sidewall verticality, squareness, and top surface roughness.
4. Repeatability and reprocessing effects: To increase the processing depth of micropillars, the processing focus was lowered after repeated processing of a single layer. The variations in the micropillar morphology with the number of single-layer repetitions and the number of times that the focus was lowered were analyzed.

## 4. Experimental Results and Discussion

Laser-induced thermal effects produced a region with color change on the C face of SiC. Optimal microscopy observation of this region indicated that the effective single-pass spot diameter was approximately 32 μm. This study determined the optimal overlap conditions along the *X*-direction and *Y*-direction to establish an appropriate parameter set for planar (areal) processing. The optimized planar parameters were then employed for 3D microstructure fabrication and subsequent characterization of the surface morphology. The corresponding experimental results are discussed in the following sections.

### 4.1. Influences of Scan Line Spacing and Scanning Speed on the Surface Morphology of SiC

**Figure 4:**
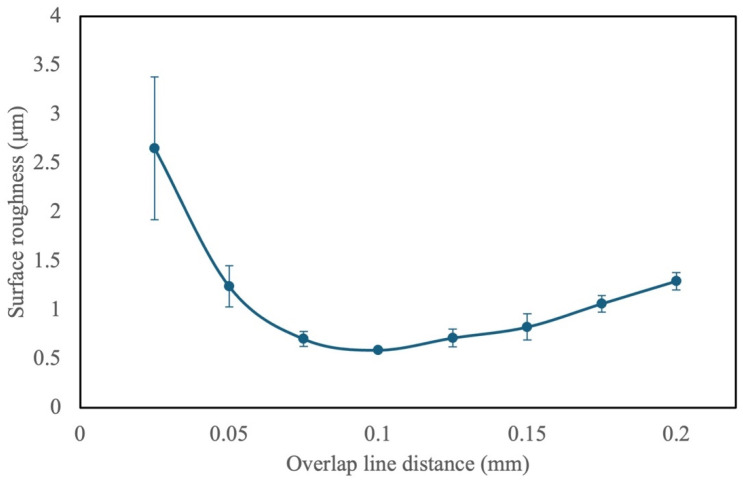
Surface roughness of SiC processed under various scan line spacings in the *Y*-direction.

The influence of the scan line spacing in the *Y*-direction on the surface roughness after processing was investigated by varying this spacing from 0.025 to 0.2 mm. As displayed in Figure 4, the surface roughness of SiC exhibited a U-shaped dependence on the line spacing. When this spacing was increased, the roughness initially decreased rapidly, reached a minimum value of 0.59 μm at a line spacing of 0.1 mm, and then increased.

Converting the line spacing of 0.1 mm into the actual processing scale (by downscaling it by a factor of 5) yielded an effective hatch spacing of approximately 0.02 mm (0.1 mm/5 = 0.02 mm = 20 μm). On the basis of the optically observed effective spot diameter of approximately 32 μm, the corresponding spot overlap ratio in the *Y*-direction was estimated to be approximately 37.5%, which is within an appropriate overlap range for areal processing. Under this overlap ratio, adjacent scan lines sufficiently covered the wafer surface and effectively suppressed residual stripe- or ridge-like features between neighboring tracks, thereby reducing geometric undulations arising from the discrete scanning trajectories. Moreover, this overlap level avoided the excessive local reheating that would otherwise occur with overly dense line spacings, which can promote heat accumulation and the formation of resolidified recast layers, ultimately increasing surface roughness.

When the line spacing was too large, that is, 0.15–0.2 mm, the corresponding actual hatch spacing increased to approximately 30–40 μm. Such large separation led to insufficiently processed regions between adjacent scan paths, resulting in periodic striping or areas in which the laser spot did not adequately overlap, which increased surface roughness. On the basis of the aforementioned observations, a line spacing of 0.1 mm (corresponding to an actual hatch spacing of 20 μm) was selected as the optimal condition. This spacing provided a favorable balance between uniformity of coverage and the suppression of heat accumulation and recast redeposition, yielding the lowest surface roughness. The aforementioned spacing was used in the subsequent multilayer fabrication of 3D microstructures.

**Figure 5:**
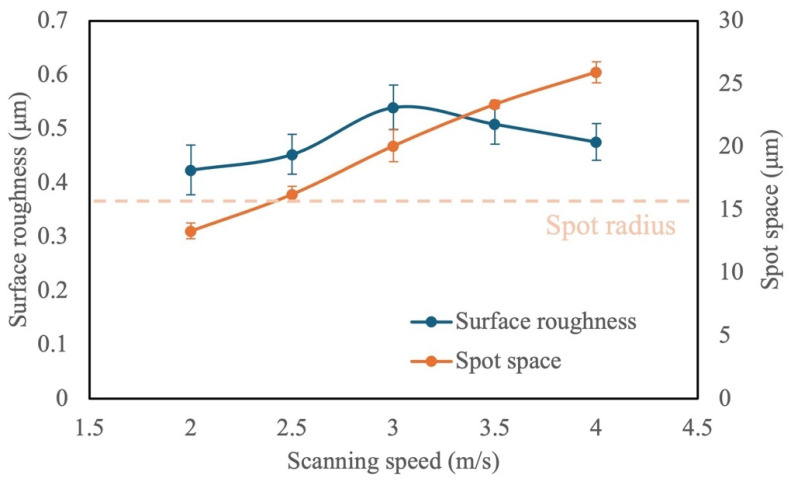
Surface roughness and laser spot space under various scanning speeds.

The scanning speed in the planar processing (*X*-direction) was systematically optimized by examining its effects on the surface roughness and spot spacing. When a focusing lens with an effective focal length of 109 mm was used, the effective spot diameter on SiC was approximately 32 μm; thus, the spot radius was approximately 16 μm. The experimental results indicated that the spot spacing increased with the scanning speed (Figure 5). At a scan speed of 2.5 m/s, the spot spacing was approximately 16.25 μm, which was the closest value to the spot radius of all spot spacings and corresponded to a pulse-to-pulse overlap of approximately 50% along the scan direction. Under this condition, the pulses overlapped appropriately and thus effectively covered the surface and smoothed out the periodic undulations and redeposited material associated with single-pulse processing, resulting in low surface roughness. Moreover, this setting prevented excessive pulse accumulation at low scan speeds, which would have enlarged the molten pool and increased the number of resolidified layers.

The surface roughness first increased and then decreased with an increase in the scanning speed. The roughness was relatively low in the low-speed regime (2.0–2.5 m/s) and slightly higher under a scanning speed of approximately 3.0 m/s. At scanning speeds of approximately 3.5–4.0 m/s, the roughness was marginally lower than that at a speed of 3.0 m/s. This trend was attributable to an increase in the scanning speed causing a decrease in the effective number of pulses delivered to a given location and a reduction in the accumulated laser dose. Consequently, the formation of melt-flow-induced ripples, resolidified particulates, and recast layers was mitigated, leading to a modest reduction in surface roughness. To achieve an appropriate balance between processing continuity and the suppression of redeposition-related defects, a scanning speed of 2.5 m/s was selected for the subsequent fabrication of multilayer 3D microstructures.

### 4.2. Influences of Laser Power and Pulse Repetition Frequency on the Surface Morphology of SiC

**Figure 6:**
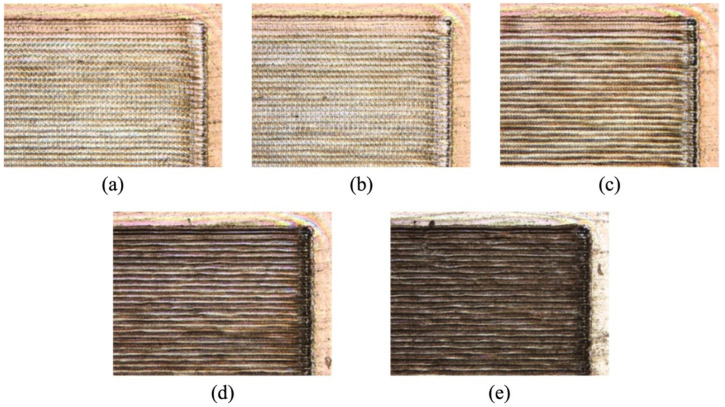
Optical images of SiC processed under a laser power of 10% and pulse repetition frequencies of (**a**) 10, (**b**) 20, (**c**) 50, (**d**) 80, and (**e**) 100 kHz.

**Figure 7:**
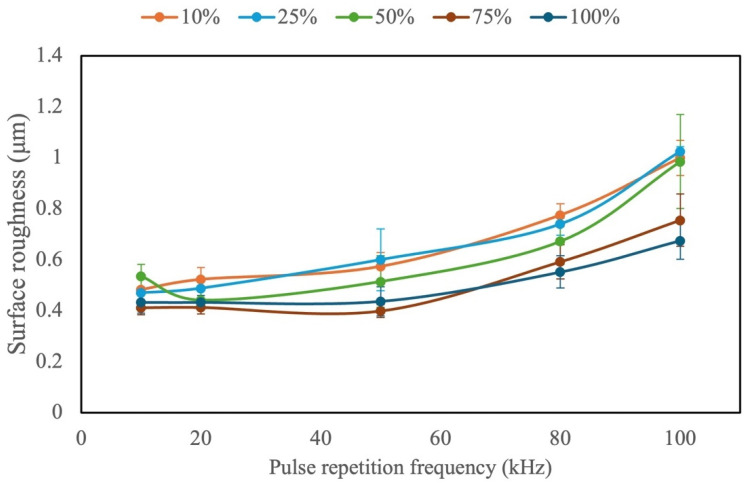
Surface roughness of SiC processed under various laser powers and pulse repetition frequencies.

The combined effects of laser power and pulse repetition frequency on ablation depth (average step height) and surface roughness were examined by fixing the hatch spacing in the *Y*-direction and the scanning speed. Figure 6 displays the optical micrographs obtained under various pulse repetition frequencies at a fixed laser power of 10%. The processed regions became progressively darker as the pulse repetition frequency was increased. This darkening was likely related to the shorter interpulse interval at higher frequencies, which prevented the surface from cooling completely between successive pulses, thus leading to higher baseline surface temperature. An increase in the surface temperature in the ambient atmosphere caused near-surface compositional redistribution and promoted oxidation. In addition, a higher repetition frequency led to more molten material and a thicker resolidified recast layer. The surface roughness and the prominence of fine-scale textures also tended to increase with the repetition frequency (Figure 7). Overall, these chemical and morphological changes reduced the surface reflectance, leading to the darker appearance in the optical images.

Figure 7 indicates that at a fixed pulse repetition frequency, the surface roughness decreased with increasing laser power. A plausible explanation for this result is that at low power, SiC processing proceeded near the ablation threshold, which resulted in nonuniform material removal and localized residual pits, leading to relatively high surface roughness. This effect was particularly pronounced when low power (10–50%) was combined with high repetition frequency (100 kHz), under which conditions the processing regime was incomplete. In this regime, the surface was substantially thermally modified and accumulated a considerable amount of recast material without exhibiting efficient material ejection, causing the roughness to approach approximately 1 μm. By contrast, when the power was higher than an effective processing threshold (i.e., 75–100%), the removal regime was more stable. Under such conditions, more continuous ablation and local remelting facilitated a smoothing effect (remelting-induced smoothing), producing a more uniform surface with lower roughness than that produced at lower power (roughness of ~0.65–0.75 μm).

Overall, the results suggested that increasing the pulse repetition frequency changed the surface response from relatively clean material removal toward a thermally dominated regime characterized by heat accumulation, melting, and resolidification, thereby increasing surface roughness. Conversely, increasing the laser power shifted the process from an incomplete processing, nonuniform modification regime to stable material removal, enabling a reduction in surface roughness at a given pulse repetition frequency.

**Figure 8:**
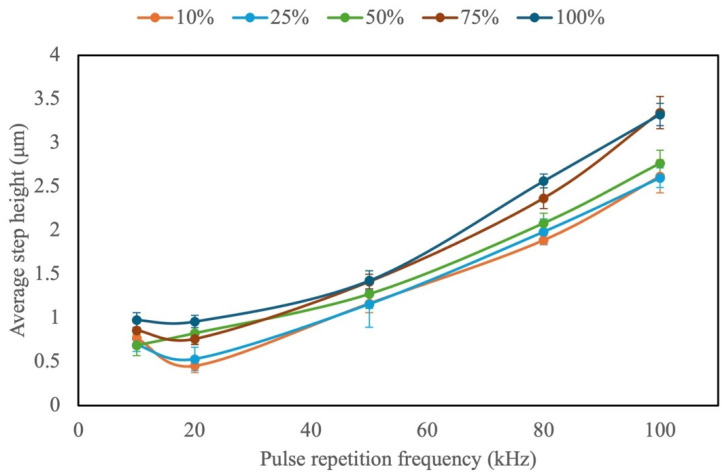
Average step height of SiC processed under various powers and pulse repetition frequencies.

Figure 8 indicates that the ablation depth (average step height) at a pulse repetition frequency of 20 kHz was lower than that at 10 kHz for all laser power values. A possible mechanism for this result is that increasing the pulse repetition frequency from 10 to 20 kHz substantially reduced the pulse energy. The higher single-pulse energy at 10 kHz more easily exceeded the SiC removal threshold, leading to more pronounced material ejection and measurable material removal. However, when the repetition frequency was increased to 20 kHz, the single-pulse energy decreased to nearly half its original value, becoming insufficient to efficiently remove material from the surface. Nevertheless, the near-surface temperature increased to the melting range, which promoted oxidation. Therefore, the molten material was not effectively removed but instead accumulated and resolidified, particularly near the feature edges, resulting in melt backfilling and a decrease in the net removal height. The aforementioned phenomena suggests that 20 kHz may represent a processing transition from an ablation- and ejection-dominated regime to a melting-and-backfilling-dominated regime. As the repetition frequency was increased beyond 50 kHz, the number of pulses that accumulated per location increased, and heat accumulation increased considerably. In this regime, the material was weakened by the progressive accumulation of defects, such as dislocations and microcracks, and the formation of an amorphous layer. In addition, multipulse irradiation altered the near-surface chemistry through oxidation and surface roughening, which enhanced optical absorption and effectively lowered the threshold for material removal (the incubation effect). Consequently, although the single-pulse energy was lower at a higher repetition frequency, the larger accumulated pulse count and lower effective material removal threshold facilitated more frequent exceedance of the effective removal condition, thereby leading to a larger net removal depth. Accordingly, at a fixed power level, the average step height increased with the repetition frequency when this frequency exceeded 20 kHz. Furthermore, at a fixed repetition frequency, the removal depth increased with increasing laser power.

**Figure 9:**
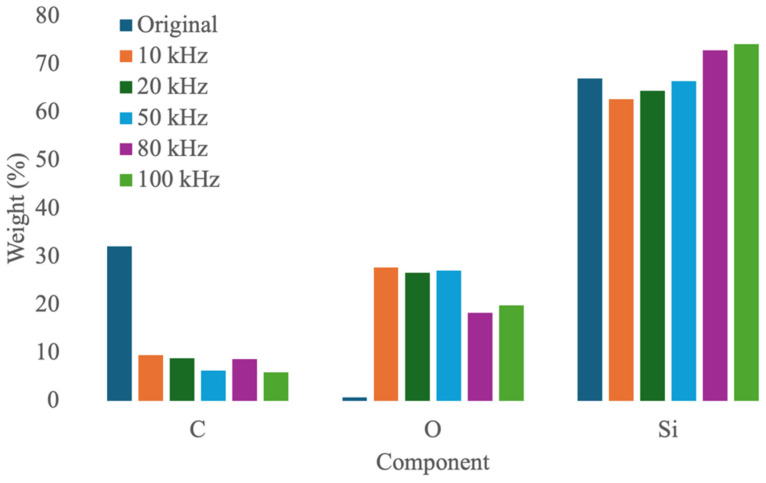
Ingredient analysis of SiC processed under power of 10% and various pulse repetition frequencies.

Next, SEM (Scanning Electron Microscope) (Regulas 8240, Hitachi, Ltd., Tokyo, Japan) and EDS (Energy-Dispersive X-ray Spectroscopy) were used to observe more microstructural characterizations and explore the corresponding mechanisms. The EDS results provide further insights into the mechanism potentially underlying the lower material removal depth observed at a pulse repetition frequency of 20 kHz. The unprocessed SiC surface primarily consisted of C (approximately 32 wt.%) and Si (approximately 67 wt.%), with only a negligible amount of O (Figure 9); these proportions align with those of the original SiC substrate. After nanosecond laser processing, all pulse repetition frequencies resulted in a decrease in C content and an increase in O content, indicating that the laser–material interaction involved not only physical ablation but also high-temperature melting, carbon loss, and surface oxidation. The influence of pulse repetition frequency was characterized by three distinct stages. In the first stage, covering 10–20 kHz, the postprocessing concentrations of C, O, and Si remained relatively similar. However, as shown in Figure 8, removal depth was lower at 20 kHz than at 10 kHz. As the pulse repetition frequency increased, the energy per pulse decreased, potentially reducing the volume of material removed through effective ejective ablation. Consequently, the 20 kHz frequency represented a critical transition regime dominated by oxidation and melting rather than efficient material ejection. At 50 kHz, the C content decreased further while the O content stabilized at approximately 26–27 wt.%; however, the machining depth began to increase. This result indicated that significant surface oxidation persisted and that the higher pulse density enhanced the cumulative effects. At this stage, several processes occurred simultaneously, including thermal decomposition of SiC, carbon loss, surface oxidation, melting and resolidification, and progressive removal of previously modified layers by accumulated laser pulses. In the 80–100 kHz regime, the material removal depth substantially increased despite an approximately 7–8-wt.% decrease in O content. With these parameters, the pulse density, effective accumulated pulse number, and accumulated laser dose were sufficiently high to allow subsequent pulses to remove the oxide or molten modified layer formed at the surface. Because the rate of oxide accumulation may be lower than the subsequent ablation rate, the O content measured by EDS decreased. Meanwhile, the accumulated laser energy remained sufficient to interact with the modified surface, resulting in a continued increase in material removal depth. These results indicate that ablation depth is not governed solely by the extent of oxidation. Instead, it depends on whether the molten material can be effectively expelled from the processing region and whether the accumulated pulses are sufficient to continuously remove the oxidized and modified layers. 

### 4.3. Influences of Pulse Repetition Frequency and Laser Power on the 3D Morphology of SiC

**Figure 10:**
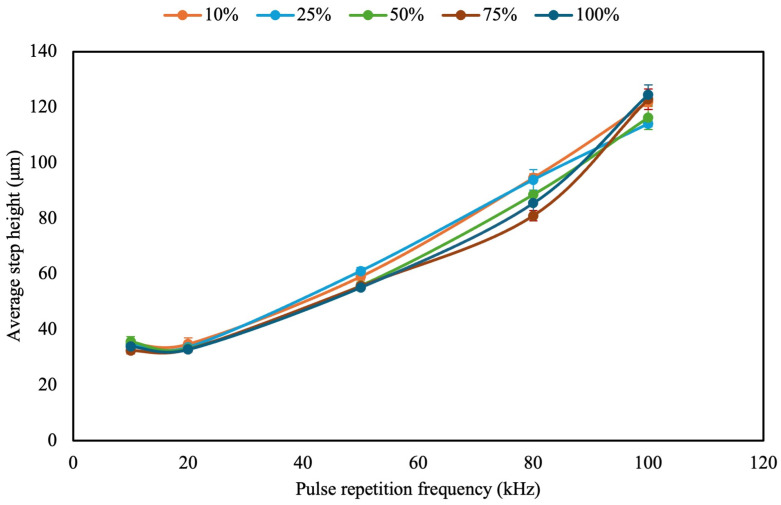
Average step height of 3D microstructures fabricated under various laser powers and pulse repetition frequencies.

After the optimal planar processing parameters were determined, 3 × 3 micropillar arrays were fabricated for validation and geometric characterization. The target array width and scan line spacing were both set as 750 μm. To ensure that the fabricated microstructures had the target dimensions, the dimensions of all arrays were prescaled by a factor of 5; thus, the scaled array width on the SiC surface was 150 μm (750/5 = 150 μm). A total of 30 consecutive layers were synthesized to achieve 3D fabrication. In single-layer planar processing, the average removal depth at a repetition frequency of 10 kHz was approximately 1 μm under various power settings. Therefore, during multilayer processing, the focal position of the focusing lens was automatically shifted downward by 1 μm after the fabrication of each layer. After the fabrication process was completed, the machining depth was quantified as the average height difference between the reference surface and the region surrounding the fabricated micropillars. This height difference did not exhibit a pronounced dependence on the nominal laser power that was set using the user interface for file loading, pattern drawing, and parameter setting (Figure 10). This was likely attributable to the following factors. First, the laser power was set as a percentage, which may not have directly scaled linearly with the pulse energy received by the workpiece. In particular, when the laser power was varied from 10% to 100%, the nominal change in laser energy was tenfold; however, the pulse energy and the efficiency of its coupling to the sample surface may have been affected by the laser characteristics and optical losses, which likely reduced the apparent sensitivity of the measured step height to the laser power. Second, because the region surrounding the micropillars included both the ablated area and regions affected by melt redeposition or resolidified material, the measured average step height reflected the combined effect of material removal and deposition, thereby masking the direct effect of laser power on the ablation depth. In addition, the mechanism and depth trend of laser pulse frequency affecting the material removal depth of 3D micropillars are the same as those of planar machining. The ablation depth (average step height) decreased marginally when the repetition frequency was increased from 10 to 20 kHz but then increased with the repetition frequency beyond a repetition frequency of 20 kHz.

**Figure 11:**
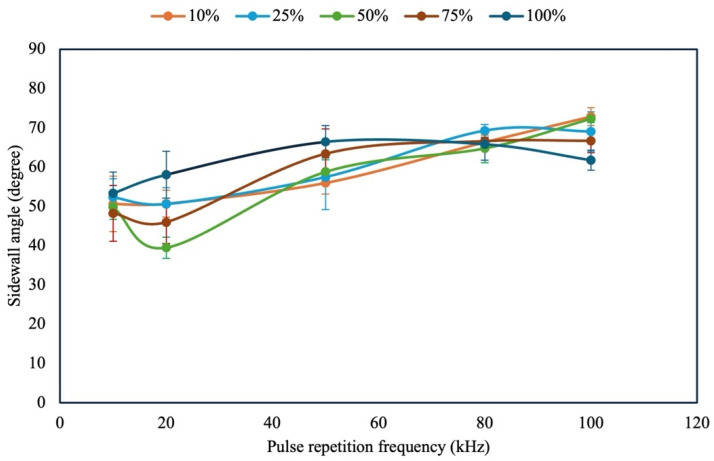
Sidewall angles of 3D microstructures fabricated under various laser powers and pulse repetition frequencies.

**Figure 12:**
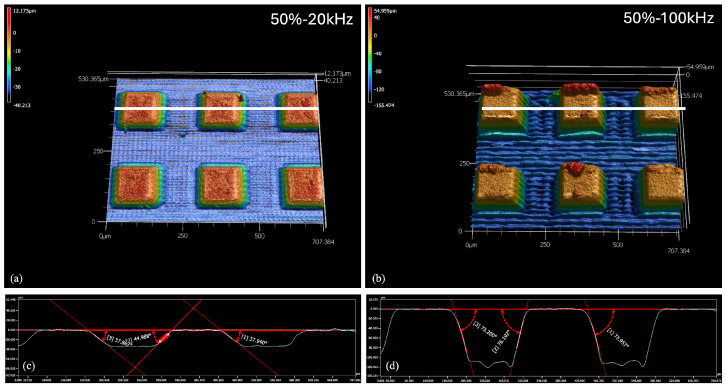
(**a**,**b**) 3D images and (**c**,**d**) profiles of 3D micropillars fabricated under a laser power of 50% and pulse repetition frequencies of 20 and 100 kHz.

Under relatively low laser power (≤75%), the sidewall angle increased with the pulse repetition frequency (Figure 11). A larger sidewall angle corresponded to higher verticality, implying that the micropillars were more upright and well-defined. Notably, at 100 kHz, the sidewall angle was approximately 75°. By comparison, at low repetition frequencies, the sidewall angle values exhibited a wider spread, particularly near the critical processing threshold around 20 kHz. For example, under a laser power of 50%, the sidewall angle was smaller than 40°, resulting in a pronounced tapered (sloped) profile [Figure 12c]. These observations suggested that lateral material removal, localized edge chipping, and nonuniform melt backfilling were more likely under lower-frequency processing, leading to lower verticality and process stability. Moreover, the influence of the laser power on the sidewall angle was weaker than that of the repetition frequency. Under high-frequency conditions (80–100 kHz), the sidewall angles for all laser power values were within the narrow range of approximately 65–75° [Figure 12d], which indicated that a high repetition frequency provided a stable processing window and enabled the formation of well-controlled 3D geometries.

The underlying causes of the aforementioned experimental results are summarized as follows. The higher single-pulse energy under a lower pulse repetition frequency induced melt formation and spatter more easily. Consequently, edge chipping and corner breakage were more likely to occur along the sidewalls, and molten material accumulated and resolidified on the sidewall surfaces more easily, producing a less straight, tapered profile. By contrast, at higher repetition frequencies, which enhanced laser energy absorption and thereby reduced the effective processing threshold. Consequently, 3D micromachining was more continuous and efficient, facilitating preferential removal of the bottom region surrounding the micropillars while suppressing lateral erosion. Thus, the sidewall angle and micropillar verticality increased. Moreover, during multilayer 3D processing, the focal position progressively shifted from layer to layer (Figure 2) such that the sidewall geometry may have been influenced by cumulative defocus as well as by melt backfilling and recast layer saturation. Accordingly, increasing the nominal laser power did not necessarily yield a proportional increase in sidewall verticality or removal depth, which reduced the apparent differences attributable to power variation. By contrast, the pulse repetition frequency directly altered the pulse count delivered per unit length and enhanced heat accumulation and thus had a stronger influence on the resulting geometric profiles than did the laser power.

### 4.4. Influences of the Repetition Frequency on the Surface Morphology and Properties of SiC

**Figure 13:**
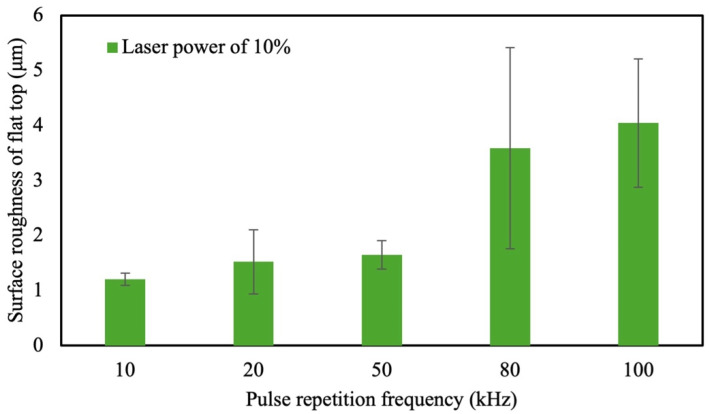
Roughness of the top surface of 3D micropillars fabricated under a laser power of 10% and various pulse repetition frequencies.

A laser power of 10% and a pulse repetition frequency of 100 kHz yielded the largest sidewall angle (i.e., the greatest sidewall verticality). Under this condition, the machining depth after the fabrication of 30 layers was approximately 120 μm. However, material spatter and subsequent backfilling around the processed region led to high surface roughness of the top surface of the micropillars (Figure 13). Therefore, experiments were conducted under a fixed laser power of 10% to examine the effects of the repetition frequency, number of fabricated layers, number of scans per layer, and focus shift on the roughness of the top surface of the fabricated 3D micropillars, their sidewall verticality, and the material removal depth. The corresponding processing strategies and parameter settings are summarized in Table 4.

**Figure 14:**
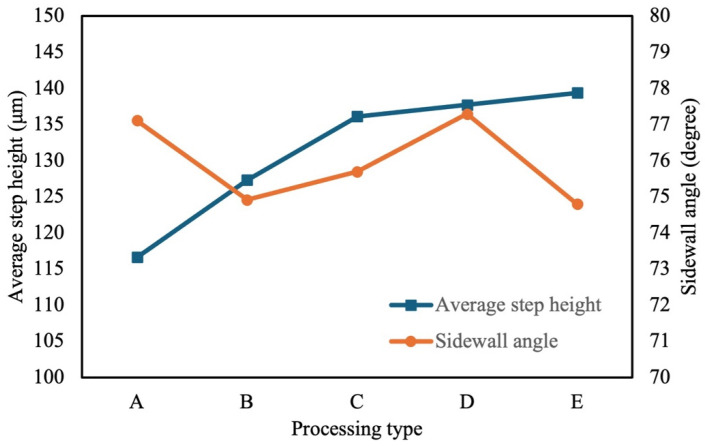
Average step heights and sidewall angles of 3D micropillars fabricated under various processing settings.

As detailed in Table 4, settings B–E involved the same total number of scans (120 passes in total) but different numbers of repetitions within each layer, various focal shifts along the *Z*-direction, and different numbers of fabrication layers. The experimental results indicated that the overall material removal depth increased progressively from setting A to setting E. When the repetition frequency and number of scans were fixed at 10 kHz and 120 (settings B–E), respectively, the material removal depth increased with the number of scans per layer and the focal shift (Figure 14). This trend was attributable to the cumulative defocus errors arising from a mismatch between the programmed focal step increment and the actual single-layer removal depth. Although the planar processing experiments suggested an average single-layer removal depth of approximately 1 μm, the effective layer-by-layer removal depth during multilayer processing is not necessarily constant and can be influenced by melt backfilling and redeposition. Consequently, focal adjustments had to be made extremely frequently when the focal plane was shifted downward by 1 μm after every pass under setting B (across 120 layers). Such frequent focal shifts might have resulted in a defocused state with larger light spots and lower energy density, thereby reducing the removal efficiency and limiting the processing depth. By contrast, when multiple scans were conducted at the same focal plane before the focus was shifted (settings C–E), the effective pulse count and accumulated energy dose in the plane were higher, which meant that the removal depth per focal plane exceeded 1 μm more easily. Therefore, subsequent focal shifts remained closer to the optimal focus condition, mitigating cumulative defocus and improving the overall material removal efficiency and total material removal depth. Of all settings considered in this study, setting E involved the fewest focal shifts and the largest energy dose in each focal plane, rendering it least susceptible to defocus accumulation. Consequently, the maximum machining depth was achieved under setting E. By contrast, setting A yielded the smallest machining depth because the total number of scan passes was only 30. Compared with the other settings (120 passes), setting A involved a higher repetition frequency, which led to a greater per-pass material removal depth due to stronger heat accumulation and incubation effects; however, the lower pass count in setting A resulted in smaller overall material removal depth.

In terms of sidewall verticality, larger sidewall angles and thus greater verticality were discovered under settings A and D than under the other settings, including setting E, which led to the largest machining depth. Thus, increasing the material removal depth does not necessarily lead to an increase in the sidewall angle; therefore, a trade-off between machining depth and sidewall angle (or quality) exists in practical applications. A plausible explanation for this finding is that increasing the number of scans within a single layer can introduce two competing effects. First, repeated scanning can promote more continuous material removal, reduce uneven lateral erosion caused by local nonachievement of material removal thresholds, and enable more stable material removal in the bottom region surrounding micropillars. Therefore, the sidewall angle increased with the number of scans per layer (from setting B to setting D), reaching its maximum value under setting D. Second, excessive scanning within a layer can increase the local thermal load and the quantity of molten material, leading to adverse effects such as melt accumulation and backfilling along the sidewalls, enhanced redeposition, and degradation of the geometric profile. Consequently, the sidewall angle under setting E was smaller than that under setting D.

**Figure 15:**
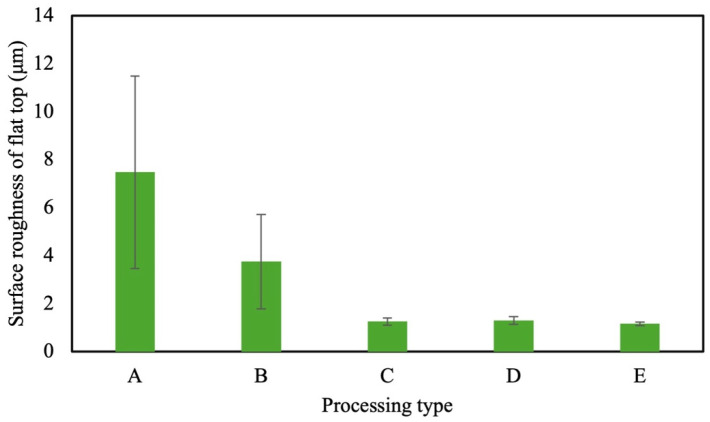
Roughness of the flat top surfaces of 3D micropillars fabricated under various processing settings.

A high pulse repetition frequency (100 kHz) was employed in setting A, resulting in a high pulse density at each processed location and a strong incubation effect. Thus, material was continuously removed, and lateral erosion was suppressed, meaning that the sidewall angle remained relatively large. However, spatter and melt backfilling also occurred, which led to a rough micropillar top surface (Figure 15). Accordingly, of all the settings, the highest top-surface roughness and largest variability in this roughness were observed under setting A, indicating that spatter and backfilling under high-frequency processing are stochastic in nature and thus less repeatable and stable. The top surface of the micropillars fabricated under setting B also exhibited high roughness (Figure 15) because of the gradual accumulation of fine redeposited particles or oxide layers; however, the surface roughness values under setting B were considerably lower than those under setting A. As the number of processing layers was decreased, the instability associated with frequent layer-wise focal adjustments and the repeated cycle of debris generation and backfilling was mitigated, leading to less particle accumulation on the flat top surface of the fabricated micropillars. Moreover, when multiple scans were conducted within each layer, the bottom region surrounding the micropillars could be removed more effectively, leading to the formation of a more distinct trench and causing a portion of the debris to be swept away by subsequent scans; thus, the likelihood of backfilling was lower. These effects contributed to the more stable processing observed under settings C–E than under the other settings. Under settings C–E, the top-surface roughness was to approximately 1 μm.

**Figure 16:**
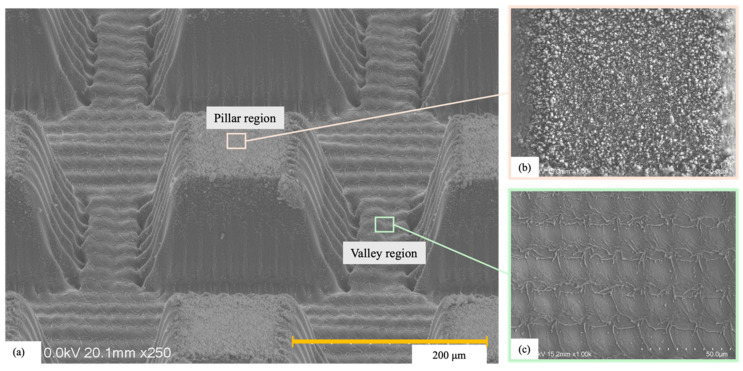
SEM images of 3D micropillars fabricated by processing type of D. (**a**) whole 3D micropillar, top view of (**b**) pillar and (**c**) valley region.

**Figure 17:**
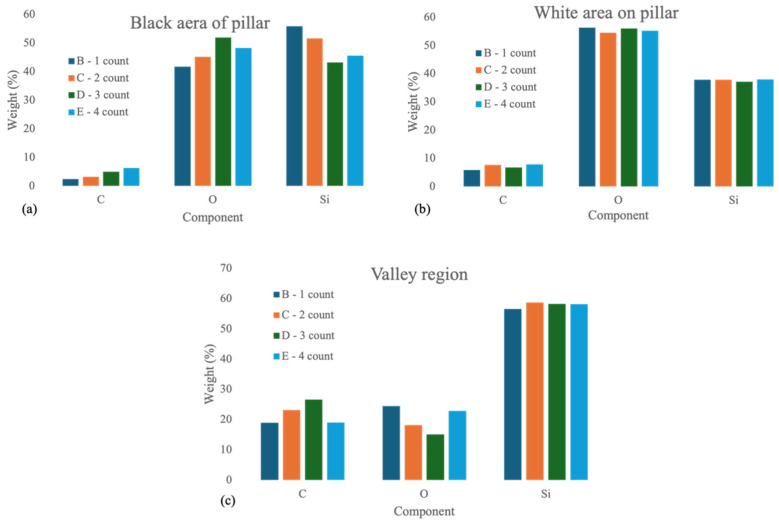
Ingredient analysis of 3D micropillars at (**a**) black and (**b**) white area of pillar top and (**c**) valley region fabricated by processing type of D.

The influence of the number of repeated scans per layer on the formation mechanism of 3D micropillars was also investigated using SEM and EDS. As shown in Figure 16, the top surface of the micropillars was divided into regions: a top region containing white spherical spattered particles and darker heat-affected areas and a bottom region containing groove structures with clear evidence of melt flow and resolidification. Quantitative compositional analysis revealed that the white particles exhibited low C and high O contents in all processing strategies, with the O content remaining at approximately 54–56 wt.% (Figure 17). This finding suggests that these particles originated from Si-rich molten material ejected from the processing region, oxidized in the air, and redeposited onto the micropillar’s top surface. Because the chemical composition of the white particles varied only slightly between the different processing strategies, the variation in top-surface roughness was likely governed by the number, size, and spatial distribution of the deposited particles rather than by changes in their chemical composition. By contrast, the composition of the bottom groove region markedly changed with the number of repeated scans per layer. When the number of scans per layer increased from one to three, the O content decreased from approximately 24 to 15 wt.%, whereas the C content increased from approximately 19 to 26 wt.%. These results indicated that the oxidized, molten, and recast modified layer formed during the first scan was progressively removed by subsequent scans performed at the same focal plane, thereby enabling more complete material removal before the next focal shift. Therefore, increasing the number of repeated scans per layer not only reduced cumulative defocus caused by frequent focal adjustments but also improved the removal efficiency of oxidized modified layers and backfilled material. This consequently increased the total machining depth and reduced spatter deposition on the micropillar’s top surface. However, when the number of repeated scans per layer was further increased to four, the O content in the bottom region increased again while the C content decreased. This finding indicates that excessive local thermal accumulation may promote melting, oxidation, and recast-layer formation. This phenomenon may explain why increasing the number of repeated scans per layer can increase the machining depth even when sidewall verticality becomes saturated or slightly deteriorates. Overall, these results indicate that an optimal range exists for the number of repeated scans per layer. Among the conditions investigated, three repeated scans per layer provided the most favorable balance among modified-layer removal, suppression of cumulative defocus, and control of local thermal loading. 

## 5. Conclusions

This study demonstrated that a 355 nm nanosecond laser can be directly used to fabricate tunable planar microstructure patterns and 3D micropillar arrays on SiC through a consumable-free process. It systematically establishes the relationship between ultraviolet nanosecond laser parameters and the morphology of three-dimensional SiC micropillar arrays, rather than being limited to conventional planar ablation or cutting. Its experimental results indicated that the processing quality of the fabricated planar and 3D microstructures was governed by the spot overlap ratio, pulse (or energy) accumulation, melt backfilling, and redeposition. For planar processing, a *Y*-direction hatch spacing corresponding to a designed scan line distance of approximately 0.1 mm (yielding an effective overlap ratio of ~37.5%) mitigated the formation of recast layers caused by overly dense scan line spacing. Moreover, an *X*-direction scanning speed of 2.5 m/s resulted in excellent surface flatness and processing continuity. For 3D fabrication, a pulse repetition frequency of 20 kHz may represent a transitional regime in which the dominant processing mechanism changes from ejective ablation to melting and backfilling, resulting in shallower removal depths than those achieved at other pulse repetition frequencies. As the repetition frequency increases beyond 20 kHz, the pulse density increases and the strength of the incubation effect increases, contributing to a decrease in the effective processing threshold and an increase in the material removal depth. Although higher repetition frequencies are beneficial for improving sidewall verticality, they can also increase the top-surface roughness of fabricated micropillars by causing spatter-induced backfilling. In addition, even under the same total number of scan passes, the number of repetitions per layer and the focal-shift strategy significantly affect cumulative defocus, total removal depth, and sidewall quality. Consequently, the optimal strategy involves a low repetition frequency (10 kHz), low nominal laser power (10%), repeated scans within each layer, and a small number of focal-shift layers. This approach enables fabrication under a relatively low local thermal load, thus preventing degradation of the integrity of sidewalls and achieving a smooth pillar top surface. With the aforementioned approach, a top-surface roughness of approximately 1 μm can be achieved while maintaining a sidewall angle of up to 77° without mask and subsequent dry-etching refinement. Overall, this study conducted a comprehensive analysis to determine the relationships between various laser processing parameters and microstructure dimensions in the direct-write fabrication of SiC micropillars. Its findings can serve as a foundation for the rapid manufacturing of heat dissipation microstructures for power devices, and further connecting 3D geometry, liquid flow behavior, and heat transfer efficiency in the future. Therein, this method is more suitable for non-active regions, sacrificial substrates, and independent SiC heat sinks, but it may not be suitable for the active regions of the device or low-defect sintered interfaces after back metallization. In the future, it may even be possible to integrate post-processing, cleaning, photo-etching, or other hybrid processes to improve sidewall verticality and surface integrity for precision application. 

## Figures and Tables

**Table 1:** Comparison of the innovative aspects between reference and this study.

Item	ICP/RIE	Femtosecond Laser	Nanosecond Laser (This Study)
Process Positioning	High-precision, vertical sidewall, wafer-level parallel machining	Low thermal damage, high-precision direct-write machining	Low-cost, maskless, rapid prototyping and thermal structure direct-write
Equipment Costs	High	Medium	Low
Photomask Requirements	Requires photoresist/hard mask	No mask needed	No mask needed
Estimated Total Process Time	Approximately 1–2 days	Approximately several hours to 1 day	Approximately 4–16 h
Post-Processing Requirements	Mask removal, cleaning, surface leveling/metallization	Cleaning, CMP, light surface etching	Ultrasonic/wet cleaning, oxidation removal, CMP, post-processing finishing
Suitable Applications	High-precision microchannels, requiring high-verticality structures	Low-damage, high-precision SiC microstructures	Non-active regions, SiC heat spreader rapid prototyping, low-cost heat dissipation microstructures
Disadvantages/Limitations	High cost, long process, complex mask preparation, and slow deep SiC etching	High equipment cost; large-area, high-depth processing is still limited by throughput	Concerns about thermal damage, roughness, and redeposition

**Table 2:** Difference in innovative aspects between reference and this study.

Reference	Main Task
[15]	Feasibility of SiC MEMS microstructure fabrication and optimization of PRF/machining revolutions.
[17]	The effect of nanosecond laser direct-write etching on surface oxidation and hardness of SiC.
[18]	Nanosecond laser-induced SiC surface modification, chemical composition, and EDS mapping.
This study	This study explores the effects of parameters such as pulse frequency on the fabrication depth, sidewall perpendicularity, and flat-top roughness of micropillar arrays, and proposes differences in the control of 3D structure quality between the number of layers and single-layer repetition.

**Table 3:** Laser specifications and experimental parameters.

Parameter	Value
Wavelength (nm)	355
Maximum laser power (W)	20
Y direction overlap rate (%)	50
Scanning speed (m/s)	2.5
Laser power (%)	10, 25, 50, 75, 100
Pulse repetition frequency (kHz)	10, 20, 50, 80, 100

**Table 4:** Micropillar machining parameters for various experimental settings.

Type	Power (%)	Pulse Repetition Frequency (kHz)	Setting Layer	Repeat Count of Each Layer	Focus Shift (μm)
A	10	100	30	1	1
B	10	10	120	1	1
C	10	10	60	2	2
D	10	10	40	3	3
E	10	10	30	4	4

## Data Availability

All data of the present study are provided in this article. Further inquiries may be directed to the corresponding author.
